# Preparation and immunopotentiating activity of a novel oil-in-water nanoemulsion adjuvant based on trimethyl chitosan

**DOI:** 10.1371/journal.pone.0359159

**Published:** 2026-10-27

**Authors:** Zhuyun Liu, Weilu Guo, Lei Wang, Pengcheng Zhao, Shanshan Yang, Donghua Zhai, Baochao Fan, Lizhong Wang, Bin Li

**Affiliations:** 1 School of Intelligent Pharmaceutical Manufacturing & New Chemical Engineering Materials, Taizhou Poly-technic College, Taizhou, Jiangsu, China; 2 School of Pharmacy, Nanjing University of Chinese Medicine, Nanjing, Jiangsu, China; 3 Institute of Veterinary Medicine, Jiangsu Academy of Agricultural Sciences, Key Laboratory of Veterinary Bi-ological Engineering and Technology, Ministry of Agriculture, Jiangsu Key Laboratory for Food Quality and Safety-State Key Laboratory Cultivation Base of Ministry of Science and Technology, Nanjing, China; Icahn School of Medicine at Mount Sinai Department of Pharmacological Sciences, UNITED STATES OF AMERICA

## Abstract

**Background:**

Subunit vaccines offer excellent safety profiles but often suffer from limited immunogenicity, requiring potent adjuvants to elicit robust protection. We hypothesized that combining a positively charged biopolymer, trimethyl chitosan (TMC), with a nanoemulsion (NE) would create a synergistic delivery system to enhance immune responses. This study aimed to formulate a TMC-loaded nanoemulsion (TMC-NE) and evaluate its potential as a safe, effective adjuvant for subunit vaccines.

**Methods:**

TMC was synthesized via the quaternization of chitosan. Its modification and cyto-compatibility were verified by FT-IR, Zeta potential analysis, and cytotoxicity assays. Based on the pseudo-ternary phase diagram, a stable oil-in-water blank nanoemulsion (NE) was prepared. Subsequently, TMC-loaded nanoemulsion (TMC-NE) was prepared with TMC aqueous solution as the aqueous phase. The formulation was characterized for its droplet size, polydispersity index (PDI), and viscosity, and its safety in vivo was evaluated in mice. Using ovalbumin (OVA) as the model antigen, the immune adjuvant activity of TMC-NE was evaluated in mice by intramuscular injection, and compared with the NE and a commercial similar adjuvant M902. Humoral and cellular immune responses were analyzed by measuring serum IgG antibody levels and splenic lymphocyte proliferation, as well as the expression of cytokines IL-4 and IFN-γ.

**Results:**

Chitosan was successfully quaternized, yielding TMC with low cytotoxicity. The formulated TMC-NE was clear and transparent, with a mean droplet size of 25.41 ± 2.13 nm, a PDI of 0.231, and suitable viscosity. In vivo, TMC-NE showed no significant tissue toxicity. Immunization studies revealed that TMC-NE stimulated IgG antibody levels comparable to the M902 adjuvant and significantly higher than the blank NE. Furthermore, TMC-NE significantly increased the expression of IL-4 and IFN-γ and promoted lymphocyte proliferation and T lymphocyte levels, indicating the induction of both humoral and cellular immune responses.

**Conclusions:**

The TMC-loaded nanoemulsion (TMC-NE) is characterized as a safe and effective antigen delivery system with potent immune adjuvant properties. These findings strongly support its prospective application as a novel vaccine adjuvant.

## Introduction

Vaccination establishes a critical line of immunological defense by inducing the production of pathogen-specific antibodies, representing one of the most cost-effective strategies for preventing infectious diseases [1]. Traditional whole-virus vaccines are effective as they preserve much of the pathogen’s structure, thereby mimicking a genuine infection and stimulating robust, long-lasting immunity. However, a well-documented risk associated with live-attenuated versions is the potential for the weakened virus to revert to a virulent form. In contrast, subunit vaccines, which utilize only specific pathogenic components, offer a significantly safer profile. The trade-off is that these vaccines often fail to elicit a sufficiently strong immune response on their own. This underscores the essential role of adjuvants, which are incorporated into vaccine formulations to enhance the body’s immune response. In practice, adjuvants help increase the proportion of individuals who develop protective antibodies following vaccination, reduce the amount of antigen required per dose, and can improve the vaccine’s stability and shelf life—all factors that facilitate broader and more effective vaccine deployment [2,3]. Currently approved vaccine adjuvants include mineral salts, emulsions, Toll-like receptor (TLR) agonists, saponins, and related immunostimulatory compounds. Concurrently, a diverse range of novel adjuvant formulations continues to be actively investigated in ongoing research [4].

Based on the requirements for relatively easy production, low cost, and effective antibody response, emulsion systems are commonly employed as adjuvants in animal vaccines [5,6]. An emulsion is a uniformly dispersed system typically composed of an emulsifier, a co-emulsifier, an oil phase, and a water phase. Common emulsion-based adjuvants include water-in-oil (W/O), oil-in-water (O/W), and water-in-oil-in-water (W/O/W) types [7,8]. W/O emulsions exhibit high viscosity, making vaccines formulated with them difficult to inject. Both Complete Freund’s Adjuvant (CFA) and Incomplete Freund’s Adjuvant (IFA) belong to the W/O emulsion category. While CFA can induce a high antibody titer, it contains Mycobacterium tuberculosis, which triggers a persistent inflammatory reaction leading to pain and ulceration at the injection site. Consequently, CFA is used only in vaccines for some large animals and is not approved for human use. IFA was subsequently developed to reduce toxicity by removing the bacterial components [9]. In the 1950s, IFA was first applied in animal influenza vaccine. However, when later used in human vaccines, serious side effects were still observed, leading to its approval being restricted to veterinary vaccines only [9].

O/W emulsions have emerged as a promising platform for vaccine adjuvant development. Vaccines formulated with it exhibit good fluidity and low injection resistance, significantly reducing administration difficulty. Combined with its reliable safety, this technology has gained increasing favor in recent years and has become a core focus in the development of novel immune adjuvants. The adjuvant MF59, approved for human use, is an O/W emulsion that is biodegradable, biocompatible, and possesses immunostimulatory effects [10,11]. However, as MF59 is primarily formulated with squalene, its long-term application faces challenges regarding supply chain stability, high purification costs, and environmental sustainability, particularly concerning the ecological impact of shark-derived sourcing [1–3]. While O/W emulsion adjuvants demonstrate excellent safety profiles, enhancing their immunological potency while ensuring sustainable production remains a key objective in vaccine development [12]. Recently, nanoemulsions have demonstrated promising development prospects in the field of vaccine delivery. Compared with conventional emulsions, nanoemulsions offer lower viscosity, smaller particle size, improved stability, and fewer toxic side effects [13–16].

Chitosan (CS) is a biopolymer that possesses favorable biological properties in vivo, such as low toxicity, good biocompatibility, and biodegradability [16,17]. It can effectively stimulate both humoral and cellular immune responses, making it a promising material for enhancing vaccine efficacy [17]. However, its poor water solubility limits its application [18]. Quaternized chitosan retains the original characteristics of chitosan and has the ability to open tight intracellular junctions. Today, quaternized chitosan particles are widely used in vaccine preparation. As a vaccine adjuvant, they can induce macrophages to secrete chemokines and related inflammatory cytokines in tissues, and promote the rapid accumulation of monocytes at the injection site [19]. Considering the advantages of chitosan, we chose to prepare trimethyl chitosan (TMC) and incorporate it into an O/W nanoemulsion as an aqueous phase component. We hypothesized that the incorporation of TMC into an O/W nanoemulsion would synergistically enhance both humoral and cellular immunity compared with the nanoemulsion alone. This dual-functional system was designed to leverage both the immunostimulatory properties of TMC and the delivery function of the nanoemulsion.

This study commenced with the chemical modification of chitosan, introducing quaternary ammonium groups at the C2 position amino group to produce water soluble trimethyl chitosan (TMC). While chitosan-based adjuvants [16,17], quaternized chitosan systems [20,21], chitosan-stabilized emulsions [16,22], and nanoemulsion vaccine adjuvants [5–8] have each been investigated separately, a key gap remains: no prior work has integrated low-degree-of-quaternization TMC directly into the aqueous phase of an O/W nanoemulsion to create a unified, synergistically acting adjuvant–delivery platform. Unlike chitosan-stabilized emulsions that rely on interfacial adsorption of the polymer, our TMC-NE formulation dissolves TMC homogeneously in the aqueous phase, thereby preserving the inherent nanoemulsion architecture while imparting permanent positive surface charge. Notably, the engineered TMC-NE system features an ultra-small droplet size of approximately 25 nm. In the context of vaccine delivery, such nanoscale dimensions are theoretically favorable, as particles in the 20–50 nm range have been widely reported in the literature to facilitate interstitial transport and may enhance the potential for interaction with antigen-presenting cells (APCs) in draining lymph nodes. Furthermore, by integrating the intrinsic immunostimulatory properties of TMC—which may serve as an immune-stimulating signal to promote immune activation—with the established encapsulation and sustained-release benefits of the nanoemulsion (NE), we hypothesized that this platform could function as a dual-purpose synergistic adjuvant-delivery system. Our findings demonstrate that vaccines formulated with TMC-NE significantly enhance OVA-specific IgG titers and promote global T-cell proliferation and cytokine secretion more effectively than the unmodified NE. Collectively, this study provides a rational design for a synergistic adjuvant-delivery platform that is distinct from previously reported TMC-containing vaccine systems, offering an experimental foundation for the development of high-efficacy veterinary subunit vaccines.

## Materials and methods

### Materials

Chitosan (degree of deacetylation [DD] = 90%) was purchased from Aladdin Biochemical Technology Co., Ltd. (Shanghai, China). White oil (Marcol™ 52) was obtained from ExxonMobil Chemical (Irving, TX, USA). Reagents including Span® 80, Tween® 80, PEG 400, 1,2-propanediol, and ethanol were sourced from Sinopharm Chemical Reagent Co., Ltd. (Shanghai, China). The Cell Counting Kit-8 (CCK-8) assay kit was procured from Beyotime Biotechnology (Shanghai, China). A universal tissue fixative was purchased from Wuhan Servicebio Technology Co., Ltd.(Wuhan, China). Red Blood Cell Lysis Buffer and premium-grade fetal bovine serum (FBS) were obtained from Biosharp Life Sciences (Hefei, China). Anti-CD3, anti-CD4, and anti-CD8 anti-bodies were purchased from BD Biosciences (Franklin Lakes, NJ, USA). DMEM and RPMI 1640 cell culture media were purchased from Yuanpei Biotechnology Co., Ltd. (Shanghai, China). Mouse IL-4 and IFN-γ ELISA kits were obtained from CUSABIO (Wuhan, China). The oil adjuvant M902 was obtained from Hangzhou Yisikang Pharmaceutical Technology Co., Ltd. (Hangzhou, China). M902 adjuvant is a novel composite veterinary adjuvant consisting of a mixture of pharmaceutical-grade mineral oil and small-molecule aqueous immunostimulants, formulated as an O/W nanoemulsion.

### Preparation and characterization of Trimethyl chitosan

#### Synthesis of Trimethyl chitosan.

Trimethyl chitosan (TMC) was synthesized according to a method adapted from the literature (Fig 1) [23]. Briefly, 5 g of chitosan (CS) was placed in a 250 mL round-bottom flask, and 70 mL of purified water was added. The mixture was stirred and allowed to swell for 1 hour, after which the pH was adjusted to 8.5 using NaOH solution. Separately, 10 g of glycidyl trimethylammonium chloride (GTA) was dissolved in 30 mL of ethanol, and the resulting solution was added to the swollen chitosan. The reaction was stirred at 60 °C for at least 8 h until the mixture became clear. The mixture was then concentrated by rotary evaporation at 85 °C and 35 rpm, without evaporating to complete dryness. An excess of ethanol was added to precipitate the product, and the crude solid was collected by suction filtration. The residue was washed with ethanol at least three times. Following filtration, the solid was dissolved in purified water, dialyzed for 48 h, and finally vacuum-dried to obtain pure TMC as a white solid.

**Fig 1:**
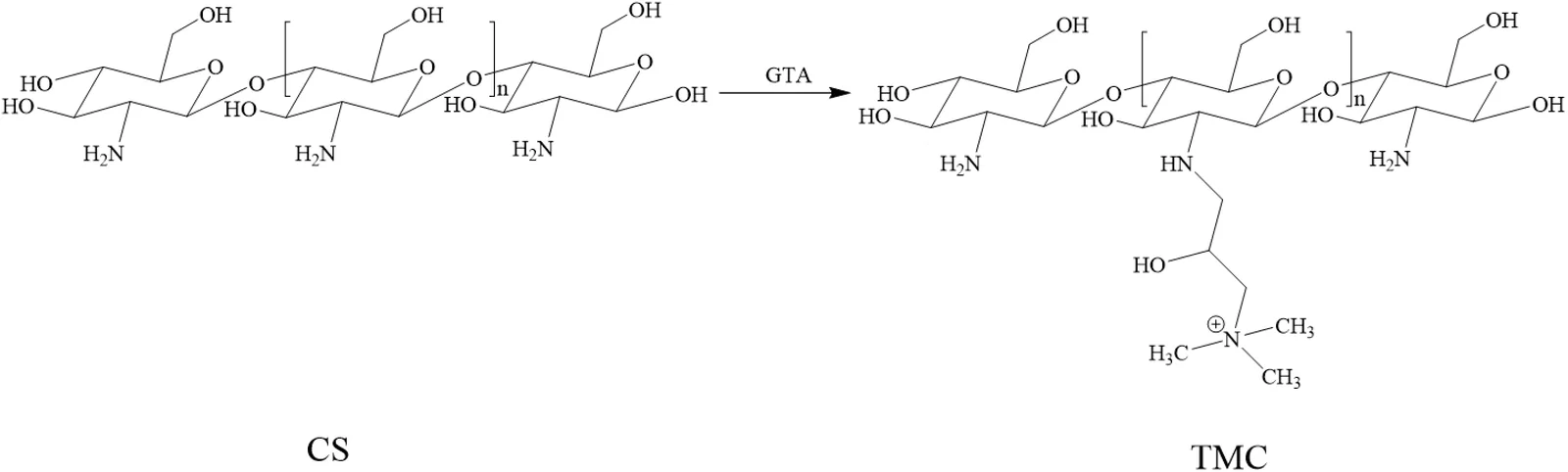
Synthesis route of TMC.

#### Fourier transform infrared (FT-IR) spectroscopy.

Fourier transform infrared (FT-IR) spectra of CS, GTA, and TMC were recorded using a Nicolet™ iS50 FTIR spectrometer (Thermo Fisher Scientific, Waltham, MA, USA). Prior to measurement, the sample stage and the pressure head were cleaned with a cotton ball soaked in ethanol and subsequently dried using an air blower. A small amount of each sample was placed with tweezers to fully cover the detector surface, and the pressure head was tightened. Overall, 40 scans per sample over a range of 400–4000 cm^-1^ were collected with a resolution of 4 cm^-1^.

#### Nuclear magnetic resonance spectroscopy (^1^H NMR).

The TMC was dissolved in D_2_O, and the ^1^H NMR spectra of TMC were obtained using an Avance 400 MHz model magnetic resonance spectrometer (Bruker, USA).

#### Cytotoxicity assay.

RAW264.7 cells (Jiangsu Hanjiang Biotechnology Co., Ltd.,Taizhou,China) were used in this study. The cells were grown to confluence, passaged and seeded into 96-well plates at a 1: 3 split ratio. Cells were cultured in an incubator at 37 °C with 5% CO_2_. TMC was serially diluted in DMEM to obtain working solutions, and 10 μL of each dilution was added to the cells in the 96-well plates to achieve final concentrations of 0, 10, 25, 50, 75, and 100 μg/mL per well. One group received 10 μL of DMEM only as an unstimulated control. After gentle mixing, plates were returned to the 37 °C, 5% CO_2_ incubator for 24 h. Then, 10 μL of CCK-8 solution was added to each well, followed by incubation at 37 °C for 2–3 h. Optical density (OD) was measured at 450 nm using a microplate reader. Cell viability was calculated according to Formula 1.


$$\begin{array}{c} \text{Cell viability }(\%)=\frac{\text{A}-\text{C}}{\text{B}-\text{C}}\times \text{1}\text{0}\text{0}\% \end{array}$$ (1)

Where: A = OD at 450 nm of the stimulated group; B = OD at 450 nm of the unstimulated control group; C = OD at 450 nm of the blank control (medium only).

### Screening of blank nanoemulsion formulations

#### Preliminary screening of nanoemulsion formulations.

Prioritizing biocompatibility and safety in nanoemulsion development, this study initially employed visual inspection to assess nanoemulsion formation. This approach allowed for the preliminary selection of formulation components, including the type of oil phase, surfactant, and co-surfactant, as well as an initial estimation of their mixing ratios. Based on experimental observations, white oil was chosen as the oil phase, Span® 80 and Tween® 80 as the surfactants. Meanwhile, 1,2-propanediol, ethanol, and PEG 400 were identified as candidate co-surfactants.

#### Screening for the optimal co-surfactant using the pseudo-ternary phase diagram method.

Using white oil as the oil phase, the optimal co-surfactant was screened by constructing pseudo-ternary phase diagrams. The candidate co-surfactants were 1,2-propanediol, ethanol, and PEG 400. Experimental parameters were fixed as follows: the cosurfactant amount was 0.8 g, and the total weight of the mixed surfactants (Span® 80: Tween® 80 mass ratio of 2: 1) was 1.2 g. During operation, surfactants and cosurfactant were premixed at room temperature. This mixture was then blended with the oil phase at gradient mass ratios ranging from 1: 9–9: 1 (oil: Smix) in separate glass vials. Under magnetic stirring, purified water was added dropwise. The system was observed until it became clear and transparent, at which point nanoemulsion formation was confirmed, and the critical water volume was recorded. Pseudo-ternary phase diagrams were generated using Origin 6.0 software (Origin Lab, Northampton, MA, USA).The size of the nanoemulsion region served as the criterion for selecting the optimal cosurfactant.

#### Screening for the optimal surfactant ratio using the pseudo-ternary phase diagram method.

Based on the results from section 2.4.2, the co-surfactant was determined and its amount fixed at 0.8 g. The total weight of the mixed surfactants (Span® 80 and Tween® 80) remained 1.2 g. To optimize the ratio between Span® 80 and Tween® 80, three gradients were tested: 1: 3, 1: 4, and 1: 5. Pseudo-ternary phase diagrams were plotted using water titration, with the area of the nanoemulsion region serving as the key cri-terion for identifying the best surfactant ratio.

#### Screening for the optimal mass ratio of surfactant to cosurfactant (Km).

Guided by the preceding results, mixed surfactants were prepared at different Km values (i.e., the mass ratio of surfactant to cosurfactant) of 1: 2, 1: 1, 2: 1, and 3: 1. The same water titration method was applied to construct pseudo-ternary phase diagrams for each ratio. The optimal Km value was selected based on the diagram exhibiting the largest nanoemulsion region.

#### Preparation and characterization of trimethylated chitosan nanoemulsion.

The formulation for trimethyl chitosan nanoemulsion (TMC-NE) was based on the previously finalized blank nanoemulsion composition. This experiment used the phase transformation method to make TMC-NE adjuvants. Specifically, weighed amounts of TMC were dissolved in purified water to prepare aqueous solutions at concentrations of 0, 1, 2.5, 5, 7.5, and 10 mg/mL, which served as the aqueous phase. Separately, the oil phase was prepared by uniformly mixing the oil, surfactant, and co-surfactant according to the blank formulation. The two phases were then combined at room temperature to produce TMC-NE at the corresponding concentrations.

The viscosity of nanoemulsions prepared with different TMC concentrations was characterized using a ViscoQC 300 rotational viscometer (Anton Paar, Austria) via a cone-and-plate system. The viscometer was equipped with a PTD100 cone-plate temperature control accessory and a CP40 rotor, which was automatically recognized via the Toolmaster™ function. For each measurement, approximately 1 mL of the emulsion sample was deposited into the center of the measurement plate using a microsyringe. The gap was precisely adjusted using the instrument’s automatic gap-setting function. The test temperature was maintained at (25.0 ± 0.1) °C with a rotational speed of 60 rpm. Once the sample temperature had equilibrated and the reading stabilized, the viscosity values were recorded in mPa·s. Each sample was measured three times in parallel. Throughout the testing process, torque values were ensured to remain within the effective range of 10%–90%. Formulations meeting the permissible viscosity requirements for vaccines were selected accordingly.

### Physicochemical characterization of TMC-NE

#### Formulation type identification by staining.

Methylene blue, a water-soluble dye that appears blue, diffuses readily in aqueous phases. Sudan red, an oil-soluble red dye, diffuses preferentially in oil phases. To identify the emulsion type, a small amount of either Sudan red or methylene blue dye was added dropwise to separate samples of freshly prepared nanoemulsion. The diffusion behavior of each dye within the emulsion was observed and compared.

#### Identification by centrifugation.

To confirm whether TMC-NE qualified as a nanoemulsion, a centrifugation stability test was performed. Freshly prepared TMC-NE was centrifuged at 5000 rpm for 30 min and inspected for signs of phase separation or breakdown, such as creaming or cracking. Samples that remained homogeneous with no visible separation were considered to meet the criteria of a stable nanoemulsion.

#### Particle size, micromorphology and zeta potential.

For morphological analysis, TMC-NE was diluted 1:100, and a droplet of the dilution was deposited onto a 300-mesh copper grid for observation by transmission electron microscopy (TEM), allowing assessment of particle morphology and size. Images were acquired using a JEOL JEM-1400plus TEM (JEOL Ltd., Tokyo, Japan). In parallel, the droplet size, polydispersity index (PDI), and zeta potential of TMC-NE droplets were determined using a Zetasizer Nano ZS90 (Malvern Panalytical, Malvern, UK). These measurements, based on dynamic light scattering (DLS) on their size distribution. Samples were appropriately diluted with deionized water prior to measurement to avoid multiple scattering effects

#### Stability study.

To evaluate the stability of TMC-NE, the samples were centrifuged at 10,000 rpm for 30 min to observe whether the system had stratified or become turbid after centrifugation. And the samples were stored at 4°C, 25°C, and 37°C for 7 days. On day 0 and day 7, the formulations were visually inspected for any physical instability, such as phase separation, precipitation, or creaming. Furthermore, the particle size, PDI, and zeta potential were recorded to monitor the microscopic stability.

The thermodynamic stability of TMC-NE was assessed through heating-cooling cycles. The formulation was subjected to two consecutive cycles, with each cycle consisting of storage at 4°C for 48 h followed by 25°C for 48 h. Following the completion of the cycles, the physical appearance was examined for any signs of instability, and the physicochemical parameters were remeasured to confirm the integrity of the nanoemulsion system.

### Animals and ethics statement

All animal procedures were approved by Jiangsu Academy of Agricultural Sciences Ethics Committee (Approval No: SYXK (Su) 2025−0026). A total of 50 female BALB/c mice (SPF grade, 6−8 weeks old, 6−20 g) were purchased from Sibefu (Suzhou) Biotechnology Co., Ltd. (Production License: SCXK (Su) 2022−0006).

The mice were housed in a facility maintained at 20–26 °C, 40–70% relative humidity, with a 12/12-h light/dark cycle. Sterilized corncob bedding was changed twice weekly. All mice underwent a 6-day acclimatization period upon arrival. Throughout the study (maximum duration: 42 days), animal health and behavior were monitored daily by personnel trained in animal care. Humane endpoints were established to minimize suffering; mice displaying severe distress (e.g., > 20% body weight loss, labored breathing, or inability to ambulate) were to be immediately euthanized. In this study, all 50 animals survived to the planned experimental endpoints without any unexpected deaths.

### Safety evaluation

#### Treatment for safety assessment.

After acclimatization, 18 mice were randomly divided into three groups (n = 6). The grouping and dosage regimen are detailed in Table 1. Body weights were recorded on days 0, 7, 14, 21, and 28 post-immunization.

**Table 1:** Group and dosage regimen for the safety assessment in mice.

Group	Component	Immunization Dose Each Mouse	Immunization Route
**G1**	Phosphate buffered saline (PBS)	100 μg	Intramuscular injection (i.m.)
**G2**	NE	100 μg	i.m.
**G3**	TMC-NE	100 μg	i.m.

#### Histopathological examination.

On day 28, all mice were euthanized via carbon dioxide (CO_2_) asphyxiation for tissue collection. The CO_2_ flow rate was carefully controlled to displace 30% to 70% of the cage volume per minute to minimize pain and distress prior to loss of consciousness, and death was confirmed by cervical dislocation before tissue harvesting. Major organs (heart, liver, spleen, lungs, and kidneys) were promptly excised. Tissues were fixed in 4% paraformaldehyde, embedded in paraffin, and stained with hematoxylin and eosin (H&E) for detailed microscopic examination to detect any tissue damage or abnormal inflammatory cell infiltration from the formulations. The histopathological evaluation was performed in a blinded manner using randomized coding of the tissue sections to ensure an objective assessment.

In addition, the heart, liver, spleen, lungs, and kidneys of mice were carefully dissected and weighed to calculate the organ to body weight ratio (organ coefficient). This ratio was used to evaluate the systemic impact of the formulations on the physiological status of the mice.

### Evaluation of the immunopotentiating effect of TMC-NE

#### Immunization scheme.

A total of 32 BALB/c mice were randomly divided into 4 groups (n = 8). The grouping and immunization scheme are shown in Table 2. Three immunizations were administered at two-week intervals.

**Table 2:** Group and dosage regimen for the immunogenicity evaluation in mice.

Group	Antigen	Adjuvant	Antigen Dose Each Mouse	Adjuvant:Antigen w/w	Immunization Volume Each Mouse	Immunization Route
G1	PBS				100 μL	i.m.
G2	ovalbumin (OVA)	NE	50 μg	1: 9	100 μL	i.m.
G3	OVA	TMC-NE	50 μg	1: 9	100 μL	i.m.
G4	OVA	M902	50 μg	1: 9	100 μL	i.m.

Each mouse received an immunization volume of 100 μL, the experimental groups received 50 μg of antigen per dose. As a positive control, the antigen was mixed with the commercial O/W nanoemulsion adjuvant M902 according to the manufacturer’s instructions.

Blood samples were collected via the retroorbital venous plexus under light isoflurane inhalation anesthesia on days 0, 14, and 28 to minimize distress. On day 42, terminal blood was collected via cardiac puncture under deep CO2 sedation, followed immediately by cervical dislocation to ensure euthanasia. Serum was separated and stored at −20°C for further analysis.

#### Enzyme-linked immunosorbent assay (ELISA).

Serum levels of OVA-specific antibodies were measured using an ELISA in samples collected before immunization and on days 14, 28, and 42 after the primary immunization. Briefly, each well of the 96-well plates was filled with 100 µL of PBS containing 100 ng/mL OVA protein, and incubated overnight at 4°C. Then, the plates were washed and blocked with 0.1% BSA in PBS at 37°C for 1 h. Next, the serum samples were serially diluted two-fold in PBS from 1:4,000–1:1,024,000. The diluted serum was incubated at 37°C for 1 h. After washing, horseradish peroxidase (HRP)-labeled Goat Anti-Mouse IgG (Proteintech, Wuhan, China) (diluted 1:5,000) was added to the wells and incubated at 37°C for 1 h. Following another washing step, 100 μL of 3,3’5,5’-tetramethylbenzidine (TMB) substrate was added to each well. The plate was incubated at room temperature for 10 min and protected from light. The reaction was stopped by adding 50 μL of stop solution to each well. The OD of each well was measured at 450 nm using a microplate reader. The endpoint titer was defined as the reciprocal of the highest serum dilution at which the OD value was greater than or equal to the mean OD of the negative control plus three times the standard deviation (Mean + 3 SD).

#### Flow cytometry analysis.

On day 42 post-primary immunization, all mice were euthanized by cervical dislocation and surface-disinfected by immersion in 75% ethanol for 5–10 minutes. Under aseptic conditions within a biosafety cabinet, mice were dissected to harvest the spleens. Splenocytes were then isolated. The density of the prepared splenocyte suspension was adjusted to 1 × 10⁶ cells/mL. The distribution of CD3 + , CD4 + , and CD8 + T lymphocyte subsets was determined by flow cytometry using a BD Accuri™ C6 Plus (BD Biosciences, San Jose, CA, USA) Data analysis and gate setting were performed using FlowJo software (Tree Star, Inc.).

#### CCK-8 assay.

Isolated splenocytes were diluted to a concentration of 10³ – 10⁴ cells/mL and seeded into 96-well plates at 200 μL per well. For each mouse, splenocytes were plated in quadruplicate wells. An additional well containing only culture medium without cells was included as a blank control. After allowing the cells to adhere, for the quadruplicate wells from each mouse, concanavalin A (ConA) was added to three of the wells to a final concentration of 10 μg/mL, while the remaining well was left untreated as an unstimulated control. Following 72 h of culture, 50 μL of supernatant was gently aspirated from each well of the ConA-stimulated group for subsequent cytokine detection (section 1.8.5). For the control wells, 50 μL of supernatant was gently aspirated and discarded. Then, 10 μL of CCK-8 reagent was added to each well, and the plates were incubated at 37 °C for 2–3 h. The OD at 450 nm was measured using a microplate reader. The splenocyte stimulation index was calculated according to Formula 1.

#### Cytokine measurement by ELISA.

After 72 h of ConA splenocytes stimulation, culture supernatants were collected. Interleukin-4 (IL-4) and interferon-gamma (IFN-γ) levels in the supernatants were quantified using mouse ELISA kits.

### Statistical analysis

Data are presented as mean ± SD, except for antibody titers, which are reported as geometric mean titers (GMT) with 95% confidence intervals (CI). For statistical evaluation, antibody titers were log10-transformed. Normality of data distribution was assessed using the Shapiro–Wilk test. One-way analysis of variance (ANOVA) followed by Tukey’s posthoc test was used for comparisons involving a single variable (e.g., body weight, organ coefficients, cytokine levels, splenocyte proliferation, and T-cell subset proportions among different treatment groups at a single time point). Two-way ANOVA followed by Tukey’s post-hoc test was applied to datasets with two independent variables (e.g., antibody titers across different treatment groups and time points). Homogeneity of variances was assessed using Brown–Forsythe or Bartlett’s test where appropriate. For antibody titers, different lowercase letters (a, b, c, d) indicate significant differences (*P* < 0.05) between groups. For other parameters, levels of significance are indicated as: ns (not significant, *P* ≥ 0.05), * (*P* < 0.05), ** (*P* < 0.01) and *** (*P* < 0.001). All analyses were performed using GraphPad Prism (version 10).

## Results

### Structural characterization and in vitro safety evaluation of TMC

The amino group (-NH_2_) at the C2 position of the glucosamine unit in CS preferentially attacks the less sterically hindered secondary carbon atom of the epoxide ring in GTA, leading to the ring-opening of the highly strained three-membered epoxy ring. Following the cleavage of the C-O bond in the epoxide ring, the oxygen atom abstracts a proton from the system to form a hydroxyl group (-OH). Consequently, the 2-NH_2_ group of chitosan forms a covalent bond with GTA, yielding TMC and completing the quaternization modification of chitosan. The proposed reaction is illustrated in Fig 2A. A sharp, strong peak appeared at 1480 cm ^−^ ¹ in the FT-IR spectrum of TMC, which should be attributed to the C-H bending vibration of the -N⁺(CH_3_)_3_ group. The absence of this peak in the CS spectrum confirms successful quaternization. In the CS spectrum, the peak at 1523 cm ^−^ ¹ corresponds to the -NH_2_ bending vibration. After quaternization, the peak strength disappeared, supporting the participation of -NH2 in covalent bonding. These observations confirm that we had successfully grafted quaternary ammonium groups on the CS molecule to obtain water-soluble modified chitosan TMC. As shown in Fig 2B, in ^1^H NMR of CS and TMC in D_2_O, δ 1.945 × 10^−6^ (purple region) is the peak of residual methyl protons of CS deacetylation; Compared with CS, TMC observed a very strong peak at about 3.304 × 10^−6^, indicating the presence of methyl group (-N(CH_3_)_3_) in the side chain of quaternary ammonium; The peaks at δ 2.442 × 10^−6^, 4.208 × 10^−6^ and 3.522 × 10^−6^ in the red region can be assigned to H-a, H-b and H-c. The degree of quaternization (DQ) was estimated to be about 19.1% using the peak width semi quantitatively.

**Fig 2:**
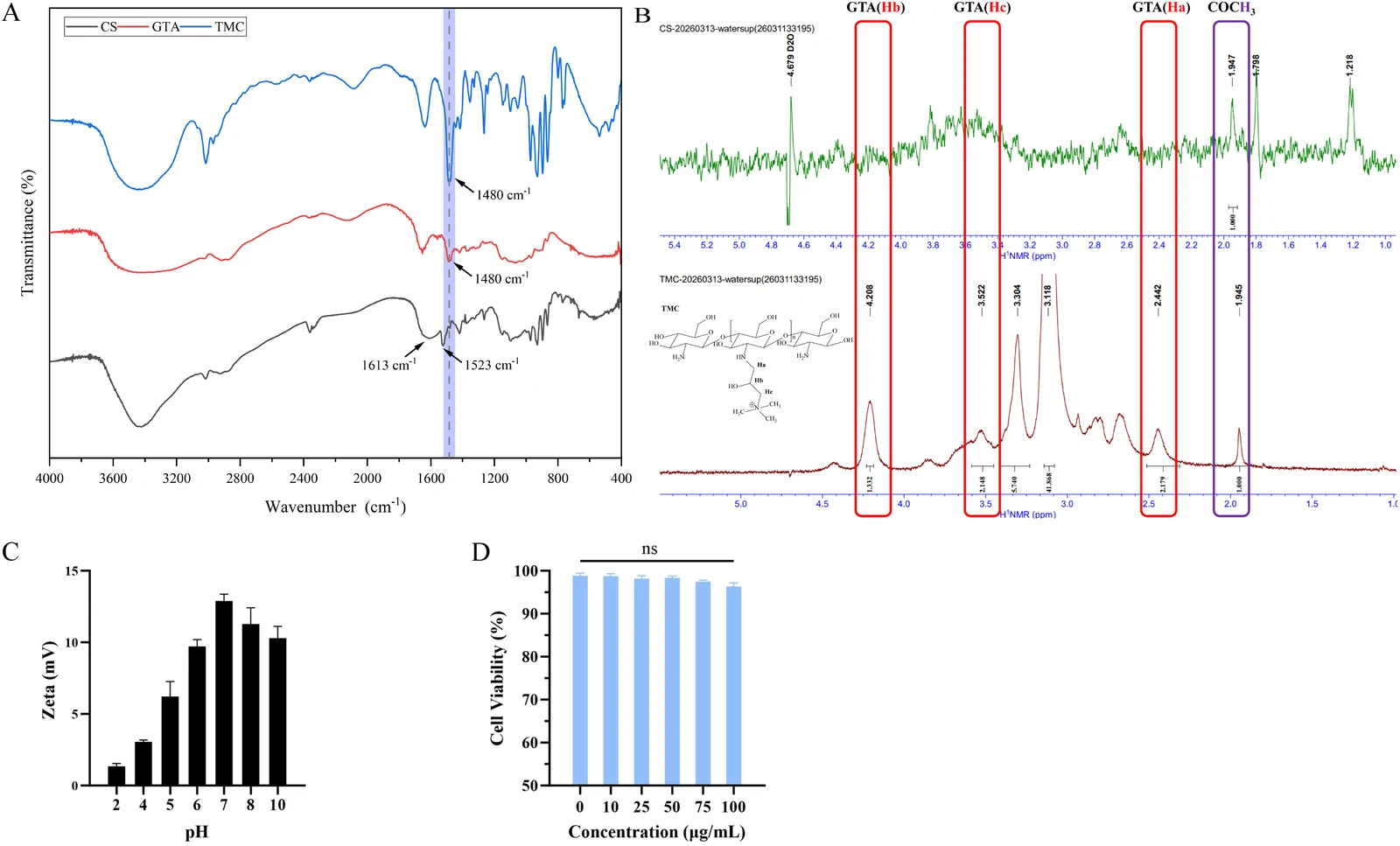
Characterization of TMC. (A) FT-IR spectra of CS and TMC. (B) ^1^H NMR spectra of CS and TMC (solvent D_2_O). (C) Zeta potential of TMC. (D) Cytotoxicity evaluation of TMC on cells. Quantitative data are presented as mean ± SD (n = 3). tatistical analysis was performed using one-way ANOVA followed by Tukey’s post-hoc test. ns indicates no significant difference (*P* ≥ 0.05).

To further characterize the material, the zeta potential of TMC was analyzed. As quaternary ammonium groups carry a positive charge at neutral pH, the zeta potential of TMC was measured across a range of pH values. As shown in Fig 2C, TMC exhibited a maximum positive charge of +12.3 ± 1.40 mV at pH 7.0, providing additional confirmation of successful quaternization.

Chitosan is inherently nontoxic. To confirm the biosafety of the modified product, the cytotoxicity of TMC was evaluated. RAW264.7 cells were treated with different concentrations of TMC, and cell viability was assessed using CCK-8 reagent after 24 h. The results are presented in Fig 2C. The results showed that the TMC-nanoemulsion system exhibited excellent biocompatibility. Within the tested range of 10–100 μg/mL, no significant cytotoxicity was observed. To further determine the safety ceiling, a wider concentration range was evaluated, yielding a calculated half maximal inhibitory concentration (IC_50_) value of 13.35 mg/mL. This confirms that both chitosan and its modified derivative, TMC, are safe and possess good biocompatibility.

### Determination of the blank nanoemulsion formulation

Guided by the principles of nanoemulsion formulation design, ethanol, 1,2-propanediol, and PEG 400 were preliminarily selected as candidate co-surfactants. Pseudo-ternary phase diagrams were constructed using the titration method, and the optimal co-surfactant was selected based on the area of the nanoemulsion region (shaded area) in the diagrams. In a pseudo ternary phase diagram, the three axes represent the aqueous phase, the oil phase, and a fixed ratio mixture of surfactant and co surfactant, respectively. As shown in Fig 3A, the nanoemulsion region was largest when PEG 400 was used as the co surfactant. Therefore, PEG 400 was chosen for the nanoemulsion formulation.

**Fig 3:**
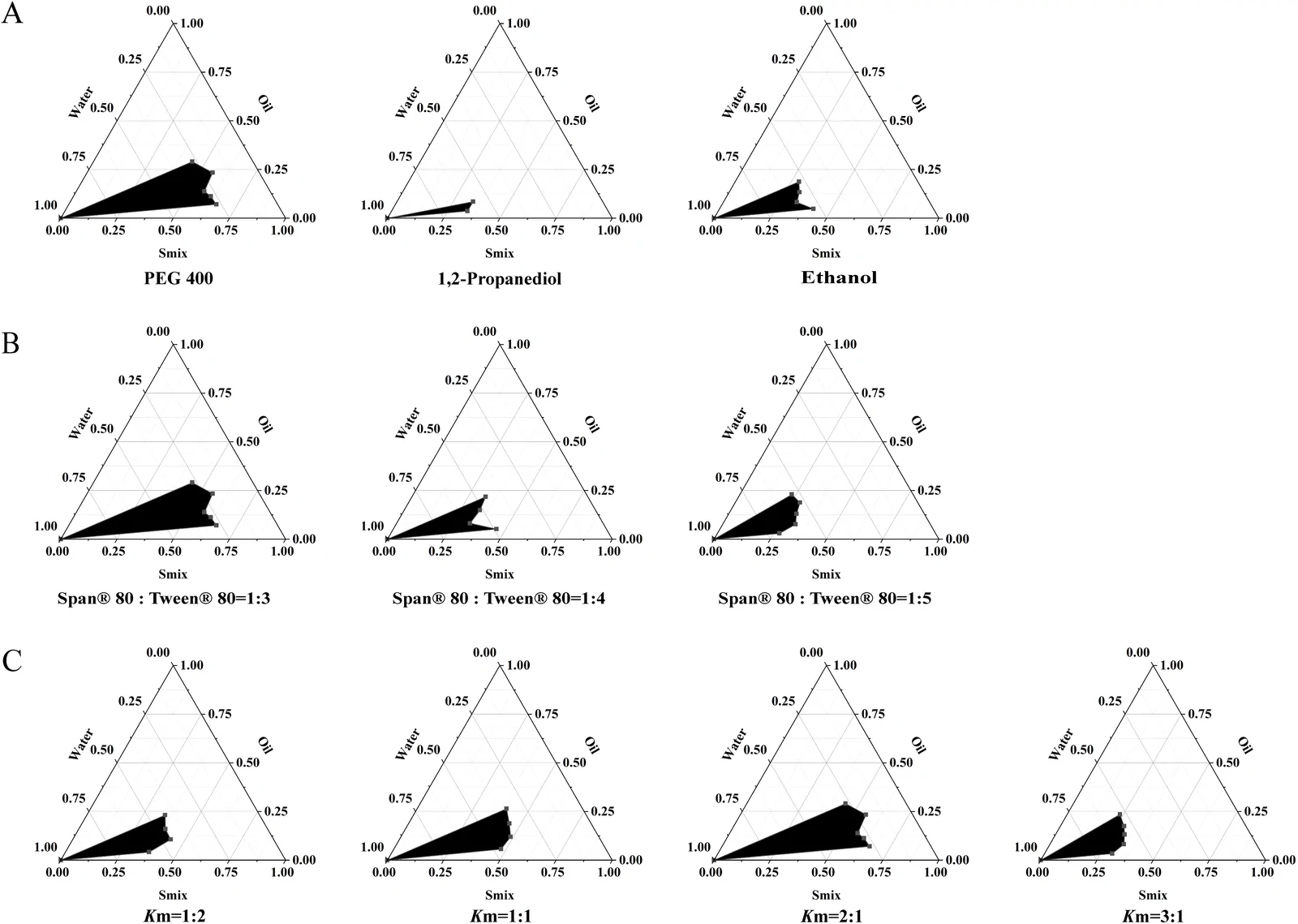
Pseudo-ternary phase diagrams for the formulation screening of blank nanoemulsion. (A) Screening of the optimal co-surfactant. (B) Screening of the optimal surfactant complex ratio ratio. (C) Screening of the *K*m value.

Pseudo ternary phase diagrams were subsequently plotted for three different mass ratios of the two surfactants, Span® 80 and Tween® 80 (Fig 3B). The area of the nanoemulsion region served as the criterion for formulation selection. Fig 3B indicates that the nanoemulsion region was maximal at a Span® 80: Tween® 80 mass ratio of 1: 3, corresponding to an HLB value of 12.325. HLB (hydrophilic-lipophilic balance) value is a measure of the surfactant’s relative affinity for water and oil; higher HLB values indicate greater hydrophilicity. The HLB of 12.325 reflects a predominantly hydrophilic surfactant mixture, which is favorable for forming oil-in-water nanoemulsions. This ratio was therefore selected as optimal.

Diagrams constructed with different *K*m values are presented in Fig 3C. The results show that the nanoemulsion area was largest at *K*m = 2: 1, which was consequently chosen for the formulation.

Based on the analysis of Fig 3, the final formulation was defined as follows: white oil: mixed surfactants: aqueous phase (O: Smix: W) = 15: 35: 50, with Span® 80: Tween® 80 = 1: 3 and *K*m = 2: 1.

### Physical characterization of TMC-NE

It is generally considered that liquids with a viscosity of less than 50 mPa·s exhibit good compliance when passing through standard needles. As shown in Fig 4A, the viscosity exceeds 50 mPa·s when the TMC level reaches 10 mg/mL. Although the viscosity at a TMC of 7.5/mL remains below the 50 mPa·s threshold, it already surpasses 40 mPa·s. Therefore, we selected a TMC concentration of 5 mg/mL for further study to ensure optimal injectability and a sufficient safety margin. The prepared TMC-NE exhibited a viscosity of 27.76 mPa·s, appearing clear and transparent with a bluish opalescence. The passage of a parallel light beam through the sample produced a distinct Tyndall effect (Fig 4B), which is characteristic of a nanoemulsion system [24].

**Fig 4:**
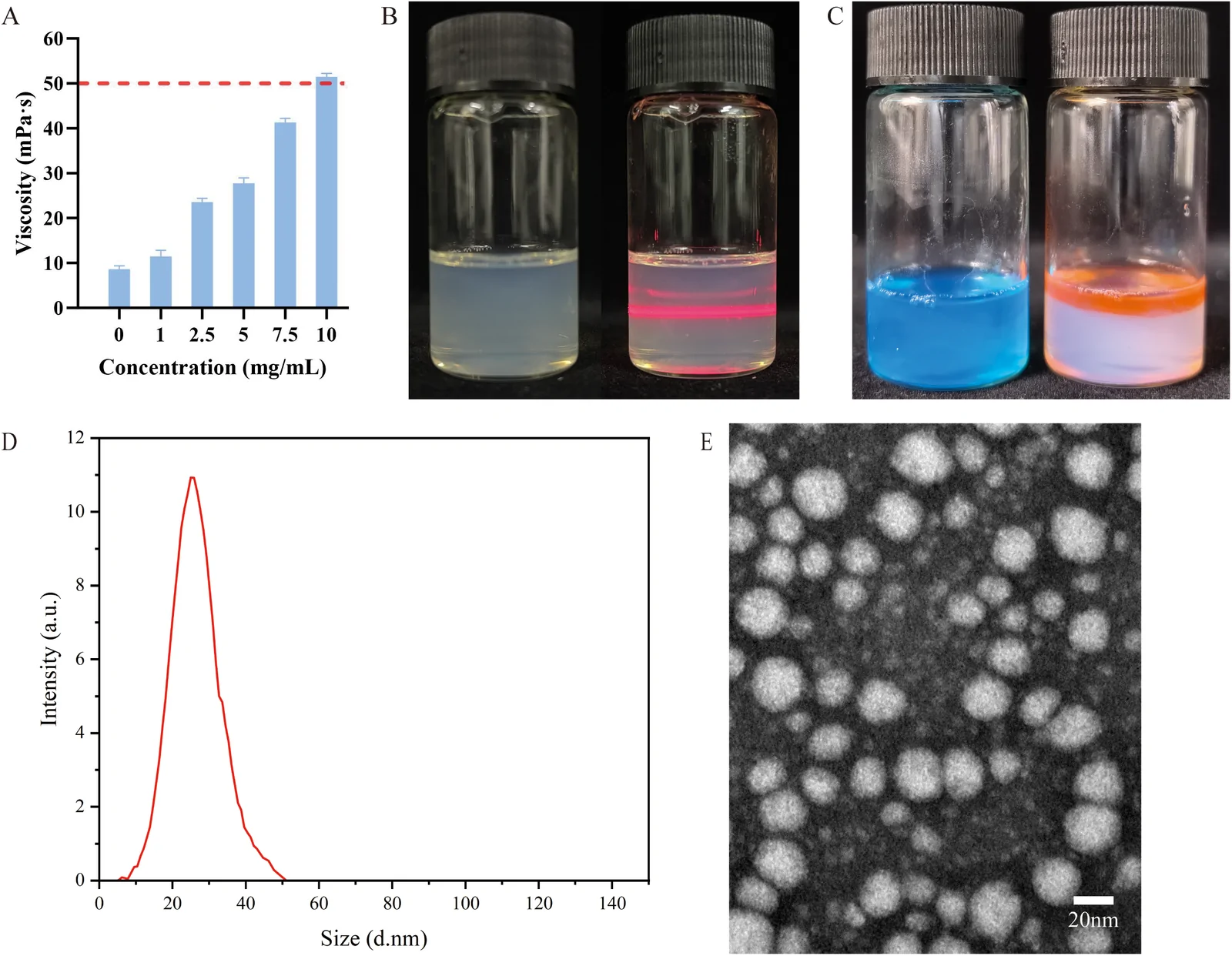
Structural characterization of TMC-NE. (A) Viscosity measurements. (B) Macroscopic appearance observation. (C) Identification of nanoemulsion type. (D) Droplets size distribution profile. (E) TEM image of TMC-NE.

In the dye diffusion test (Fig 4C), methylene blue spread more rapidly through TMC-NE than Sudan red, confirming its oil-in-water (O/W) structure. Particle size analysis by DLS showed that TMC-NE had an average diameter of 25.41 ± 2.13 nm and a low PDI of 0.231 (Fig 4D), indicating a narrow size distribution. TEM images further revealed uniformly distributed nanodroplets (Fig 4E), visually corroborating the DLS results. Furthermore, the Zeta potentials of NE and TMC-NE were measured to be −8.43 ± 0.52 mV and +3.88 ± 0.35 mV, respectively. Given that the Zeta potential of TMC alone was + 12.3 ± 1.40mV at pH 7.0, the observed charge inversion from negative to positive strongly indicates the successful incorporation of TMC into the NE droplets.

Furthermore, after centrifugation at 10000 rpm for 30 min, TMC-NE showed no phase separation or demulsification, confirming its stability as a nanoemulsion system. As shown in Table 3, the zeta potential of TMC-NE remained remarkably stable across all tested conditions. Specifically, the initial zeta potential was + 3.88 ± 0.35 mV, and no significant fluctuations were observed after 7 days of storage at 4°C, 25°C, or 37°C (*P* > 0.05). Furthermore, the zeta potential was maintained at +3.81 ± 0.39 mV after two heating-cooling cycles, suggesting that the cationic TMC remains robustly integrated with the nanoemulsion droplets under thermal and thermodynamic stress.

**Table 3:** Physical stability of TMC-NE under different storage conditions and thermodynamic stress.

Storage Conditions	Time/Cycle	Size (nm)	PDI	Zeta Potential (mV)	Visual Appearance
Freshly Prepared	Day 0	25.41 ± 2.13	0.23 ± 0.02	+3.88 ± 0.35	Clear
4°C	Day 7	25.62 ± 1.95	0.24 ± 0.01	+3.92 ± 0.35	Clear
25°C	Day 7	25.88 ± 2.04	0.23 ± 0.06	+3.75 ± 0.48	Clear
37°C	Day 7	26.15 ± 1.85	0.25 ± 0.02	+3.7 ± 0.51	Clear
H-C Cycles	2 Cycles	25.98 ± 2.10	0.24 ± 0.01	+3.81 ± 0.39	Clear

No significant difference was observed between the treated groups and the initial group (*P* > 0.05).

### Safety evaluation of TMC-NE

After intramuscular administration of NE and TMC-NE, all mice remained in good health, displaying normal locomotor activity, feeding, and drinking. No local adverse reactions, such as congestion, edema, or ulceration, were observed at the injection sites. The body weight of mice was measured on days 0, 7, 14, 21, and 28 post-injection to evaluate the effect of NE and TMC-NE on weight gain. As shown in Fig 5A, the weight gain trends across different injection groups were similar, indicating that NE and TMC-NE did not significantly affect mouse body weight. On day 28 after injection, the major organs (heart, liver, spleen, lungs, and kidneys) were collected from each experimental group and weighed. The organ-to-body weight ratio was calculated to further assess the systemic impact of NE and TMC-NE. The results (Fig 5B) showed no significant differences among the PBS, NE, and TMC-NE groups. To further investigate the biosafety of the TMC-NE adjuvant, pathological examination of key organs (heart, liver, spleen, lungs, and kidneys) was performed via H&E staining. As presented in Fig 5C, no obvious pathological alterations were observed in the tissues from any of the groups.

**Fig 5:**
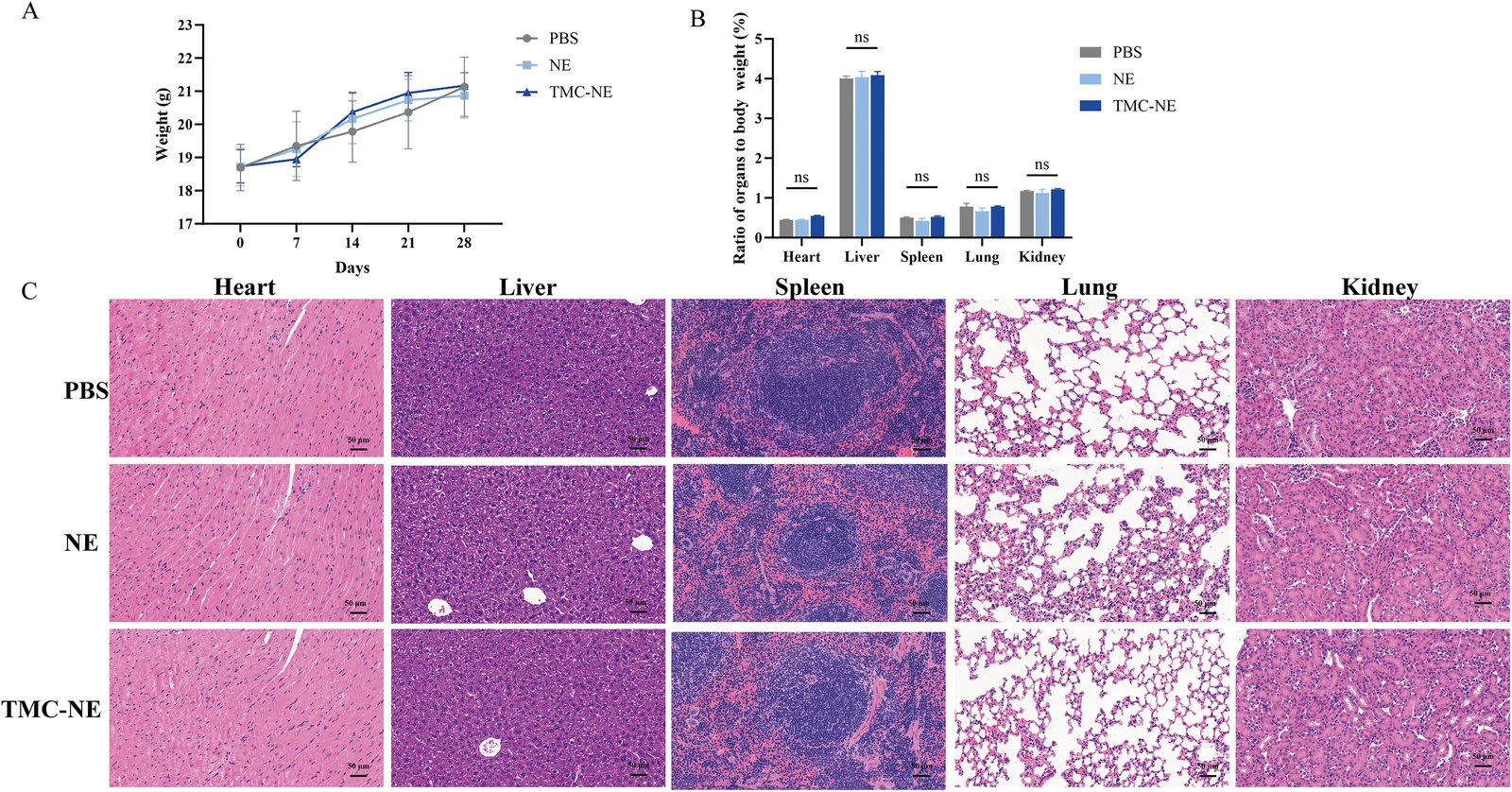
Safety evaluation of TMC in mice. (A) Body weight changes of mice over the observation period. (B) Organ coefficients (organ/body weight ratio). (C) Representative H&E staining images of major tissues(Scale bar = 50 μm; Magnification, × 40). Data are presented as mean ± SD (n = 6). Statistical analysis was performed using one-way ANOVA followed by Tukey’s post-hoc test. ns indicates no significant difference (*P* ≥ 0.05).

### Evaluation of the immunopotentiating effect of TMC-NE

Mice were immunized with vaccines prepared by formulating OVA with blank nanoemulsion (NE), TMC-NE, or the commercial oil-in-water adjuvant M902—a mineral-oil-based O/W nanoemulsion adjuvant currently approved for veterinary use in China that served as a clinically relevant benchmark. Blood was collected on days 14, 28, and 42 post-primary immunization for serum separation, and OVA-specific serum IgG levels were detected. As shown in Fig 6A, OVA-specific IgG antibody titers in all immunized groups were significantly higher than those in the PBS control group. On day 42, the OVA/TMC-NE group elicited the highest OVA-specific IgG antibody titer, reaching a GMT of 234753 (95% CI: 161929–340327). This response was significantly superior to those induced by the NE and M902 adjuvants (*P* < 0.05). These results indicate that all vaccinated mice produced high levels of antigen-specific IgG antibodies after three immunizations, and TMC-NE stimulated stronger IgG antibody responses than the M902 adjuvant.

**Fig 6:**
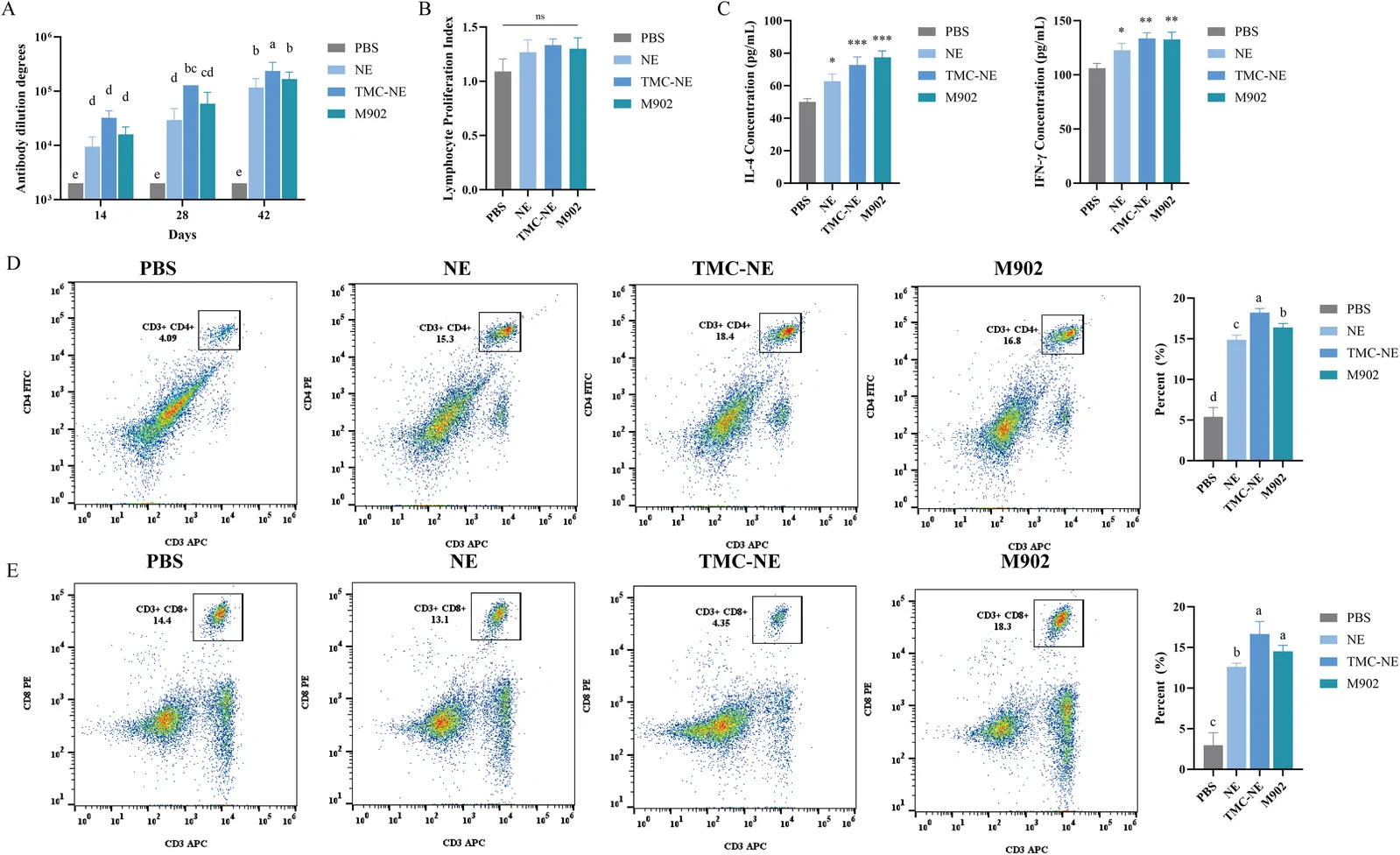
Evaluation of the immune enhancement effect of TMC-NE in mice. (A) Serum IgG antibody levels. (B) Splenocyte proliferation index. (C) Secretion levels of IL-4 and IFN-γ in spleen cells measured by ELISA. (D) Flow cytometry analysis of CD3^+^ CD4^+^ and CD3^+^ CD8^+^ T cell populations in splenocytes. Data are presented as mean ± SD (n = 8). Statistical analysis was performed using two-way ANOVA (for A) and one-way ANOVA (for B, C, D) followed by Tukey’s post-hoc test. ns indicates no significant difference (*P* ≥ 0.05); * *P* < 0.05; ** P < 0.01; *** *P* < 0.001. Groups with different letters (a–d) are significantly different from each other (*P* < 0.05).

Splenocytes isolated from immunized mice were stimulated with ConA, a non-specific T-cell mitogen. After 72 h of culture, splenocyte proliferation was assessed via the CCK-8 assay. It is important to note that ConA stimulation evaluates overall immune responsiveness and T-cell activation potential rather than antigen-specific cellular immunity. Results in Fig 6B show that all vaccinated groups exhibited greater proliferation than the PBS control. Notably, the OVA/TMC-NE group demonstrated a modestly higher proliferative response compared to the OVA/NE and OVA/M902 groups, suggesting that all experimental vaccines induced a certain level of cellular immune response, and TMC-NE elicited a slightly stronger cellular immune response than NE and M902. These results suggest that the experimental vaccines, particularly TMC-NE, systemically enhanced the overall proliferative capacity and immune readiness of lymphocytes, rather than an antigen-specific memory response.

After stimulating splenocytes with ConA for 72 h, the supernatants were collected. ConA stimulation were analyzed by ELISA for IL-4 and IFN-γ secretion. As shown in Fig 6C, secretion levels of both IL-4 and IFN-γ in all experimental groups were significantly higher than those in the PBS group upon non-specific stimulation. IL-4 and IFN-γ levels in the OVA/TMC-NE and OVA/M902 groups were significantly higher than those in the OVA/NE group (*P* < 0.05), indicating that all three vaccines could induce cytokine secretion, with M902 and TMC-NE showing better efficacy. Notably, the increase in IL-4 was more pronounced than that of IFN-γ in the M902 and TMC-NE groups, suggesting a possible bias toward a Th2-type immune response.

The proportions of CD3 ⁺ CD4⁺ and CD3 ⁺ CD8 ⁺ T lymphocyte subsets in the spleen were analyzed by flow cytometry on day 42 post-primary immunization (Figs 6D and E). All vaccinated groups showed significantly higher proportions than the PBS control. The OVA/TMC-NE group had a significantly higher proportion of CD3 ⁺ CD4 ⁺ T cells than all other groups. The proportion of CD3 ⁺ CD8 ⁺ T cells in the OVA/TMC-NE group was slightly higher than that in the OVA/M902 group, though the difference was not statistically significant. These findings demonstrate that TMC-NE effectively modulates the systemic immune microenvironment by promoting T cell differentiation in vivo. It should be emphasized that the flow cytometry data and the ConA stimulation results presented here reflect the global activation potential of the T-cell pool, which serves as indirect evidence of adjuvant efficacy, rather than direct antigen-specific T-cell memory responses.

## Discussion

This study successfully synthesized quaternized chitosan (TMC) and, for the first time, integrated it directly into the aqueous phase of an O/W nanoemulsion to construct a TMC-NE adjuvant delivery system. Unlike chitosan-stabilized emulsions in which the polymer adsorbs at the oil–water interface, or previously reported TMC-based particulate carriers, our approach dissolves TMC homogeneously within the nanoemulsion’s aqueous compartment. This design preserves the intrinsic architecture of the nanoemulsion while endowing it with permanent positive surface charge, representing a distinct class of adjuvant that combines the delivery advantages of a nanoemulsion with the immunostimulatory properties of quaternized chitosan. In an OVA model antigen system, this system demonstrated superior immune-enhancing effects compared with both the unmodified NE and the commercial benchmark adjuvant M902.

Polysaccharides have garnered increasing attention owing to their favorable biocompatibility and high safety profiles [25–36]. While many natural polysaccharides exhibit promising immunomodulatory activity, their practical application is often constrained by limited water solubility [20]. In this study, the quaternized derivative TMC exhibits excellent water solubility, allowing it to dissolve directly in a neutral aqueous phase during nanoemulsion construction. This eliminates the need to employ acidic solvents, such as glacial acetic acid, required to dissolve natural chitosan, thereby maintaining nanoemulsion stability and safeguarding the encapsulated model antigen (OVA) from conformational changes or acid-induced inactivation.

Unlike the pH-dependent protonation of native chitosan, the introduction of quaternary ammonium groups confers TMC with permanent positive charges [21]. This may help the TMC-NE system maintain a stable surface potential under physiological conditions (pH ≈ 7.4), circumventing the limitations of native chitosan which tends to undergo deprotonation and aggregation at neutral pH. While the magnitude of Zeta potential is positively correlated with the DQ, excessively high charge density may induce cytotoxicity via membrane disruption [28]. In this study, a TMC with a low DQ (approximately 19.1%) was synthesized, exhibiting high cell viability. Notably, the synthesized TMC with a low DQ imparted only a relatively low surface positive charge (+3.88 ± 0.35 mV) to the TMC-NE system. Although this zeta potential value is below the conventional threshold of ±30 mV typically associated with electrostatically stabilized colloids, the formulation nonetheless demonstrated robust physical stability, with no phase separation observed after centrifugation or under various storage conditions (Table 3). This apparent discrepancy may be explained by the steric stabilization conferred by the hydrated TMC chains extending from the droplet surface, which can prevent droplet coalescence even in the absence of strong electrostatic repulsion. Such steric stabilization has been documented for other polymer-coated nanoemulsion systems and is consistent with our stability data. Although this weak positive charge is insufficient to generate strong electrostatic attraction, it may offer distinct biological advantages. First, it could partially neutralize the negative charge of the blank NE, thereby reducing the electrostatic repulsion barrier between the nanodroplets and the cell membranes of antigen-presenting cells (APCs), which may facilitate the approach of the nanodroplets toward the cells. Additionally, this near-neutral surface characteristic could help to minimize the non-specific adsorption in vivo, potentially extending circulation time within tissues.

Regarding the potential mechanisms of cellular uptake and cross-presentation, given the relatively weak electrostatic interactions involved, one possible explanation is that the efficient endocytosis of TMC-NE could rely on receptor-mediated recognition of its polysaccharide backbone by macrophages and dendritic cells, although this remains to be experimentally verified. Furthermore, as this study utilized TMC with a low degree of substitution, retaining a substantial number of free primary amino groups on its backbone, it is plausible that it may possess TLR-recognition capabilities analogous to those of CS, which could potentially contribute to innate immune activation [29]. However, direct evidence for TLR engagement was not obtained in the present work. It has been proposed that upon entering the acidic endosome, the free primary amino groups within TMC could undergo protonation, which may facilitate endosomal escape of the antigen (OVA) into the cytoplasm, creating conditions permissive for subsequent immune activation [29]. This hypothesis is consistent with the observation by Verheul et al. that a higher degree of acetylation of chitosan—and consequently fewer free primary amino groups—was associated with a diminished adjuvant effect, underscoring the functional importance of free primary amino groups [33]. These mechanistic possibilities warrant further investigation using techniques that directly probe uptake pathways and endosomal trafficking. Studies indicate that the immune response elicited by nanoparticle vaccines is size-dependent [21,28]. Nanoparticles smaller than 100 nm can traffic within the lymphatic system and are preferentially taken up by APCs. Particles ranging from 200 to 600 nm are readily recognized by antigen-presenting cells (APCs) in the lymph nodes, eliciting a Th1-type cellular immune response. Larger particles elicit different immune responses; for example, 2–8 µm particles are presented to lymph nodes via phagocytosis or pinocytosis by macrophages, stimulating humoral immunity [31]. TMC-NE droplets showed an average size of 25.41 ± 2.13 nm. Given their small size, droplets in this range may theoretically be expected to favor a Th1-type response [32]. However, the particularly significant increase in IL-4 observed in our study suggests a bias toward a Th2-type immune response [33]. It is important to emphasize that no direct evidence is provided here that the ~ 25 nm size itself was the causal factor for the enhanced immunogenicity. The favorable immune outcomes may also stem from the TMC component and the overall nanoemulsion architecture. The Th2-skewed profile is likely attributable to OVA itself, which serves as a prototypical antigen in murine models that inherently favors the induction of Th2 responses [34–36]. Furthermore, the effective humoral immune response elicited by the TMC-NE formulation may also reflect the contribution of the nanoemulsion delivery system in promoting antigen presentation. Regarding the assessment of immune responsiveness, we observed that the TMC-NE group exhibited a stronger capacity for splenocyte proliferation upon stimulation with ConA. It is critical to reiterate that ConA is a non-specific T-cell mitogen. Therefore, the proliferation and cytokine data primarily reflect the overall functional status and activation potential of the T-cell pool, rather than definitive antigen-specific cellular immunity. Our results demonstrated that TMC-NE significantly elevated the secretion of IFN-γ and promoted splenic lymphocyte proliferation. IFN-γ is a hallmark cytokine of the Th1 response, which enhances the microbicidal activity of macrophages and stimulates the production of cytotoxic T lymphocytes (CTLs). These findings indicate that TMC-NE not only induces specific humoral immunity but may also significantly enhance the basal reactivity and immune readiness of the host’s cellular immune compartment. This augmentation of the non-specific immune background could stem from an initial sensitizing effect of low-substitution-degree TMC on immune cells, although this hypothesis requires further experimental validation. Although this study has not yet directly verified antigen-specific T-cell responses through antigen-specific stimulation assays, the observed enhancement in lymphocyte transformation capacity demonstrates the potent immunomodulatory potential of TMC-NE. Based on the cytokine profile (elevated IL-4 with concomitant IFN-γ secretion), the TMC/OVA vaccine appears to induce a mixed Th1/Th2-type response in mice, albeit with a predominant Th2 bias. Although humoral immunity (Th2) predominates, the enhanced lymphocyte proliferation indicates that the overall T-cell pool is in a highly sensitized and expanded state, which may provide a supportive cellular foundation for defense against complex infections. This consideration is relevant for evaluating whether TMC-NE, when employed as an adjuvant, could contribute to effective immune responses against intracellular pathogens. When infecting a new host cell or spreading between cells (such as during the hematogenous dissemination phase of viruses), intracellular pathogens must traverse the extracellular fluid environment [37]. At present, TMC-NE is capable of eliciting a robust humoral immune response; specifically, these induced antibodies can bind to pathogens before they contact or enter target cells, potentially preventing attachment and entry. For many veterinary or human vaccines, high antibody titers remain a key indicator for evaluating protective efficacy. Future studies employing diverse antigens beyond OVA will be necessary to determine whether the observed Th2 bias is antigen-dependent. These findings suggest that the TMC-NE platform may hold potential not only for enhancing humoral immunity but also for developing vaccine strategies that could benefit from augmented cellular immune responsiveness.

Most vaccine adjuvants do not rely on a single component to exert their immunopotentiating effects. Instead, they act through the combined mechanisms of various constituents [38–40]. M902 was selected as a comparator because it is a commercially available mineral-oil-based O/W nanoemulsion adjuvant approved for veterinary vaccines in China, representing a clinically relevant performance benchmark for our TMC-NE system. Its primary mechanism is thought to rely on the “depot effect”—slow release of antigen at the injection site with induction of localized, non-specific inflammation to recruit immune cells. In contrast, TMC-NE incorporates the immunostimulatory component TMC; this may explain why, under identical immunization protocols, the TMC-NE group achieved superior results in terms of antibody titers and cytokine levels. This outcome is consistent with a “synergistic adjuvant effect” in which both physical delivery and immuno-stimulation contribute to the overall response. Compared with other chitosan-based delivery systems, the TMC-NE platform demonstrates several distinguishing features. Previous studies have confirmed that emulsions stabilized by chitosan hydrochloride (CS-HCl) or traditional chitosan emulsions can serve as adjuvants [16,22]. However, these systems may be limited by charge instability at physiological pH or by the potential cytotoxicity associated with unmodified chitosan. As indicated by research regarding the application of chitosan derivatives in nanovaccines [41], optimizing their physicochemical properties through chemical modification is key to enhancing adjuvant efficacy.

Despite the encouraging results, this study presents several limitations that should be considered when interpreting the findings. First, although OVA served as an effective model antigen for proof-of-concept purposes, its inherent Th2-biasing nature may have influenced immune polarization. The use of a single model antigen precludes generalization to diverse pathogen-derived antigens. Future studies utilizing a broader panel of antigens from actual pathogens are required to comprehensively evaluate the broad-spectrum potential of TMC-NE. Second, the cellular immune responses were assessed exclusively via ConA-induced lymphocyte proliferation and cytokine secretion. ConA is a non-specific mitogen; therefore, these assays evaluate the global activation potential, overall immune readiness, and systemic remodeling of the T-cell pool, rather than antigen-specific memory responses. The lack of antigen-specific cellular immune assays (e.g., ELISPOT, intracellular cytokine staining, or MHC-tetramer staining) is a notable limitation that precludes definitive conclusions about the induction of antigen-specific T-cell immunity. Third, this study was restricted to a murine model, and the translational relevance to target veterinary species (e.g., swine or poultry) remains to be established. Fourth, the evaluation period was limited to 42 days post-primary immunization, providing only short-term immunogenicity data; the longevity of the induced immune responses was not assessed. Additionally, our stability assessment was confined to short-term thermodynamic stress tests; consequently, the long-term shelf life and robustness of the nanoemulsion under various storage conditions remain to be investigated. Finally, the specific molecular pathways and innate immune receptors triggered by TMC-NE have yet to be elucidated and represent important directions in future mechanistic studies.

## Conclusions

In conclusion, this study suggest that the TMC-NE system serves as a promising and biocompatible platform for enhancing immune responses against subunit antigens in a murine model. By combining the intrinsic immunostimulatory properties of TMC with the delivery advantages of a nanoemulsion, this synergistic adjuvant system demonstrated the potential to address certain immunogenicity limitations of modern subunit vaccines. The induction of balanced Th1/Th2 responses, alongside a favorable safety profile, indicates that TMC-NE is a noteworthy candidate for further development. It should be noted that the cellular immune findings reflect enhanced immune responsiveness and T-cell activation potential rather than definitive antigen-specific cellular immunity, and the mechanistic basis of the adjuvant effects warrants further investigation. These results provide a preliminary foundation for exploring its application in veterinary vaccines, specifically for economically significant species such as swine, pending further validation in target animals.

## Supporting information

S1 Fig ^1^HNMR spectra of CS and TMC. This file contains the raw ^1^HNMR spectra of CS and TMC.
(PDF)

S1 Table FT-IR data of TMC. This file contains the raw numerical data for the Fourier-transform infrared (FT-IR) spectroscopy of TMC.
(XLSX)

S2 Table Formulation screening and structural characterization data. This file contains three sheets providing the underlying numerical data for the pseudo-ternary phase diagrams, particle size distribution, and viscosity measurements of the nanoemulsion.
(XLSX)

S3 Table Safety evaluation data. This file contains two sheets detailing the raw data for the body weight changes and organ-to-body weight ratios (organ coefficients) of the mice over the observation period.
(XLSX)

S4 Table Immunological evaluation data. This file contains four sheets providing the underlying numerical data for the immunological assays, including serum IgG levels (ELISA), splenocyte proliferation index, cytokine secretion levels (IL-4 and IFN-γ ELISA), and flow cytometry analysis of T cell populations.
(XLSX)
