# Supplementary material for: Preparation and immunopotentiating activity of a novel oil-in-water nanoemulsion adjuvant based on trimethyl chitosan
Source: PLoS One. 2026 Oct 27;21(10):e0359159. doi: 10.1371/journal.pone.0359159 (PMC13712623; doi:10.1371/journal.pone.0359159)

CS-20260313-watersup(26031133195)

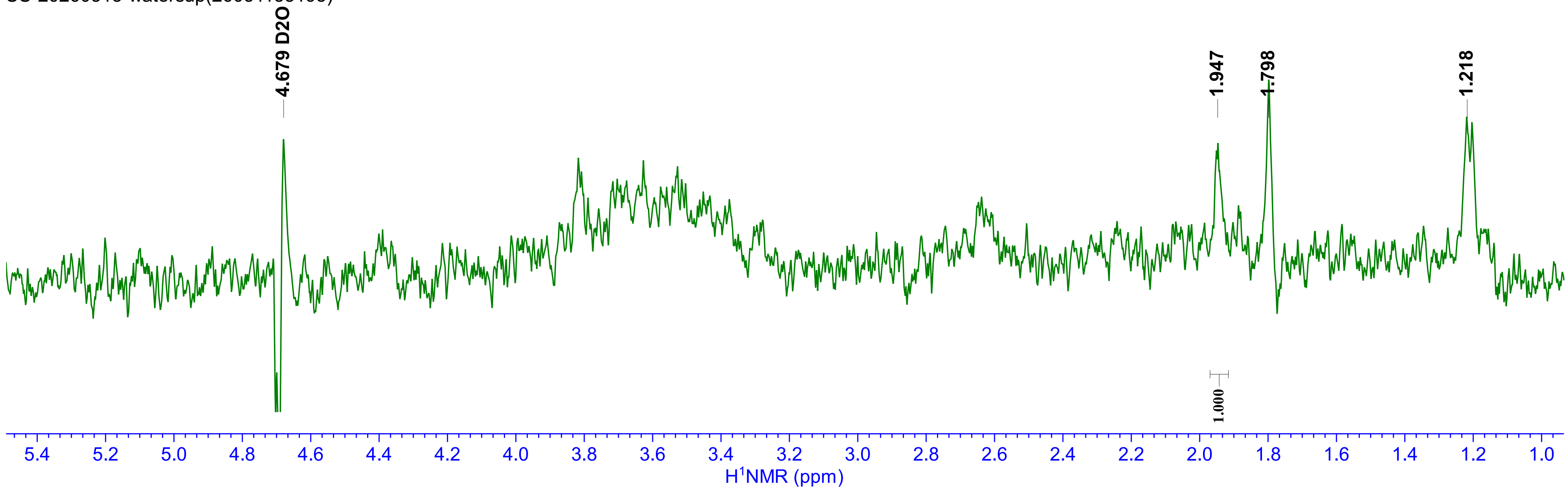

TMC-20260313-watersup(26031133195)

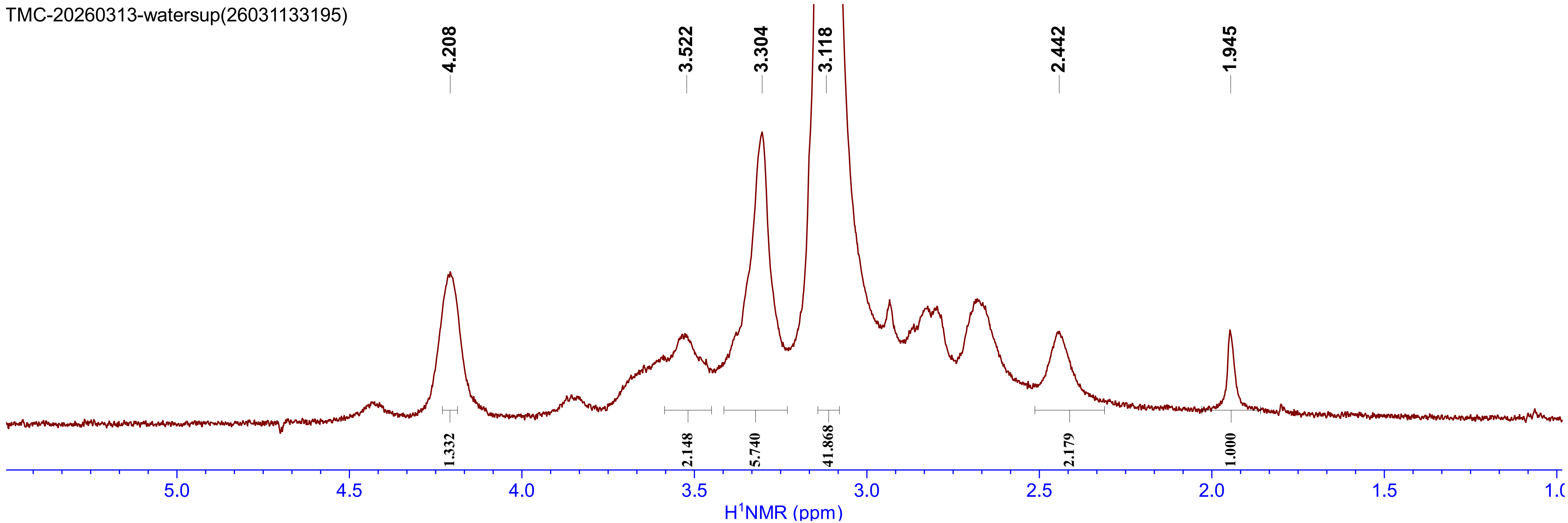

Supplement: Supplementary file 1 [file pone.0359159.s001.pdf]
